# From concept to content: assessing the implementation fidelity of a chronic care model for frail, older people who live at home

**DOI:** 10.1186/s12913-014-0662-6

**Published:** 2015-01-22

**Authors:** Maaike E Muntinga, Karen M Van Leeuwen, François G Schellevis, Giel Nijpels, Aaltje PD Jansen

**Affiliations:** Department of General Practice and Elderly Care Medicine, EMGO+ Institute for Health and Care Research, VU University medical center, Amsterdam, the Netherlands; NIVEL (Netherlands Institute for Health Services Research), Utrecht, the Netherlands

**Keywords:** Implementation fidelity, Comprehensive care model, Elderly care, Primary care, Chronic care model, Complex intervention

## Abstract

**Background:**

Implementation fidelity, the degree to which a care program is implemented as intended, can influence program impact. Since results of trials that aim to implement comprehensive care programs for frail, older people have been conflicting, assessing implementation fidelity alongside these trials is essential to differentiate between flaws inherent to the program and implementation issues. This study demonstrates how a theory-based assessment of fidelity can increase insight in the implementation process of a complex intervention in primary elderly care.

**Methods:**

The Geriatric Care Model was implemented among 35 primary care practices in the Netherlands. During home visits, practice nurses conducted a comprehensive geriatric assessment and wrote a tailored care plan. Multidisciplinary team consultations were organized with the aim to enhance the coordination between professionals caring for a single patient with complex needs. To assess fidelity, we identified 5 key intervention components and formulated corresponding research questions using Carroll’s framework for fidelity. Adherence (coverage, frequency, duration, content) was assessed per intervention component during and at the end of the intervention period. Two moderating factors (participant responsiveness and facilitation strategies) were assessed at the end of the intervention.

**Results:**

Adherence to the geriatric assessments and care plans was high, but decreased over time. Adherence to multidisciplinary consultations was initially poor, but increased over time. We found that individual differences in adherence between practice nurses and primary care physicians were moderate, while differences in participant responsiveness (satisfaction, involvement) were more distinct. Nurses deviated from protocol due to contextual factors and personal work routines.

**Conclusions:**

Adherence to the Geriatric Care Model was high for most of the essential intervention components. Study limitations include the limited number of assessed moderating factors. We argue that a longitudinal investigation of adherence per intervention component is essential for a complete understanding of the implementation process, but that such investigations may be complicated by practical and methodological challenges.

**Trial registration:**

The Netherlands National Trial Register (NTR). Trial number: 2160.

## Background

In the last decades, gerontological and geriatric researchers have been increasingly incorporating the concept of frailty into their work. In general, people are considered frail when they have entered a life stage defined by multiple health problems and loss of reserves on several domains of functioning [1–3]. As populations are aging and life expectancy is rising, the group of frail, older people is increasing. In Europe, the prevalence of frailty is estimated to range from 4.1% among middle-aged people to 17.0% among people of 65 and over [4]. Since frailty is associated with multimorbidity, disability and loss of functioning, frail, older people often have an increased need for chronic care [1]. Changes in health policy and the increasing desire to ‘age in place’ have caused the sites on which this care is delivered to relocate from the institutional setting to the home environment [5,6], making frail, older people one of the fastest growing populations in primary care [7,8]. As a result, pressure on primary and community care systems has been increasing, and elderly care is facing a number of challenges: frail older people’s health and care needs are not always identified at a timely stage, care is fragmented and coordination between care professionals is often inadequate, and older people experience a lack of involvement in their own care process [9]. These challenges necessitate a reorganization of the way in which primary elderly care is organized and provided.

With the aim to achieve this reorganization, researchers, policy makers and health care professionals have been looking to design and implement models of care delivery that relieve the pressure on primary care systems whilst upholding quality standards for elderly care. One of these models is the Geriatric Care Model, a framework for integrated care delivery based on the Chronic Care Model (CCM) [10]. The CCM aims to guide quality improvement and disease management activities in care for patients with a chronic disease. The model envisions productive interactions between a prepared, proactive practice team and an informed, activated client, and is based on the premise that the delivery of chronic care services takes place in a primary care environment that consists of three domains: the community domain, the health care system domain, and the provider organization domain. In this environment, the foundation of optimal care delivery consists of six essential elements, or ‘pillars’, of which four refer to the content of the care process (i.e. self-management support, delivery system design, decision support, and clinical information systems) and two to its context (i.e. community resources and policies and health care organization). Since the adaptability of the CCM framework allows it to be tailored to practice, in Europe, the framework has been used to guide disease management for a diverse range of patients with a chronic illness in several health care environments and settings [11–13]. However, its usefulness in integrated primary or community care settings that cater to patients with complex health problems, such as frail older people, has not been as thoroughly explored [13,14].

Although there are indications that the CCM has the potential to improve quality of care delivery, its effect on patient outcomes is uncertain. Some studies report results on outcomes such as length of hospital stay, while others find no results at all [15–17]. The inconsistencies might be due to the fact that CCM-based interventions are often complex: they involve multiple health and social care disciplines and combine elements of existing interventions. The aggregation of several interacting and potentially effective components and variations in delivery across sites complicate the relationship between intervention and impact, which challenges the interpretation of research outcomes. Insight in the true nature and delivery of CCM-based models in primary care is necessary in order to help researchers understand what factors cause such interventions to fail or succeed, what components work, and for whom [18–21].

One way to gain insight in the way in which an intervention is carried out is by assessing implementation fidelity, defined as the degree to which interventions are implemented as planned by the developers [22]. Implementation fidelity moderates the relationship between an intervention’s content and its intended outcomes. Previous studies have demonstrated that the fidelity with which a program is implemented influences its level of success [22–26]. When implementing health and care interventions, measuring implementation fidelity helps researchers understand whether a lack of success is due to inappropriate service delivery or program inadequacies, which prevents them from drawing inaccurate conclusions about program effectiveness (a so-called ‘type III error’) [27–29]. In addition, insight into the fidelity of an intervention facilitates further improvement of intervention outcomes and dissemination of research findings into practice. However, despite repeated recommendations, fidelity assessments have not yet been fully adopted as common practice in primary elderly care research [19,20,30,31].

When implementing and testing the Geriatric Care Model in primary care, we therefore aimed to assess implementation fidelity, using the framework proposed by Carroll et al. [20]. Carroll sees adherence, defined as “the degree to which implementers adhere to the intervention as intended by the intervention designers”, as the main element of fidelity, and distinguishes four adherence subcategories: content, coverage, frequency and duration. Content refers to the ‘active ingredients’ of an intervention, i.e. the essential services that an intervention aims to deliver to intervention participants. Coverage, frequency and duration quantify how much of the intervention’s content was delivered to the target group, how often and for how long. According to Carroll’s framework, all four concepts need to be evaluated to get an understanding of fidelity: the more subcategories of adherence investigated, the more ‘complete’ the assessment of fidelity. In addition, Carroll suggests that a sound assessment of implementation fidelity includes an investigation of factors that may modify fidelity. Identification of such factors contributes to our comprehension of what causes fidelity to be either high or low, and could help us overcome barriers to adequate implementation in the future. Four potential moderators are intervention complexity, availability of facilitation strategies, quality of delivery and participant responsiveness [20]; Hasson has proposed adding a fifth and a sixth moderator, i.e. recruitment and context [32].

In this paper, we present the results of a study that assessed the implementation fidelity of the Geriatric Care Model. We describe key components of the intervention program, and give insight in the degree to which professionals adhered to the delivery of these components. In order to provide an explanation for our findings, we describe moderating factors and other factors that may have influenced implementation. We hope to contribute to the existing knowledge about the implementation of the CCM in geriatric settings, and to the assessment of fidelity of complex interventions in primary elderly care.

## Methods

### The geriatric care model: objective and content

The Geriatric Care Model aims to restructure the way in which care for frail, older people in primary care is delivered. Through early identification of health and care needs, improved client autonomy and enhanced coordination between care professionals, it envisions to improve older people’s quality of care and, subsequently, their quality of life. The geriatric care model was integrated into routine practice by primary care practices, supported by practice nurses and geriatric expert teams. Geriatric teams managed practice nurses, who proactively visited older people at home and carried out a geriatric assessment. Assessment outcomes were reviewed by the nurse and a primary care physician. The Geriatric Care Model was implemented at a patient level, care professional level and organizational level by means of the older Adults: care in Transition (ACT) study [33]. The intervention and its intended three-level implementation is described below.

### Implementation at a patient level: geriatric assessment and care plan

Every six months, frail older people received an assessment of health and care and a tailored care plan. This procedure involved two home visits. During the first visit, practice nurses conducted a comprehensive geriatric assessment using the web-based Community Health Assessment version 9.1 of the Resident Assessment Instrument (RAI-CHA) [34]. RAI-CHA aims to facilitate the identification of possible health and care needs, helps users standardize their routines and works as a reminder system for follow-up. After each assessment, practice nurses wrote a care plan in consultation with a primary care physician. Care plans were based on Client Analysis Protocols (CAPs) generated by the RAI-CHA instrument. RAI-CHA items trigger CAPs in several domains (e.g. physical wellbeing, social functioning, living and safety) and help users identify possible targets for care. Approximately two weeks after the first visit, practice nurses discussed the care plan with the older person in a follow up-visit. During this second visit, nurses provided information on guideline-concordant management and treatment options, and stimulated older people to get involved in the decision making process. If necessary, an evaluation consultation was scheduled after 3 months.

### Implementation at a care professional level: quality management and education

Practice nurses were organized in care teams managed by geriatric expert teams. A geriatric expert team consisted of a geriatric nurse and an elderly care physician. In the Netherlands, an elderly care physician is specialized in the health issues of older people with complex or chronic disorders. The geriatric expert team guided and managed quality processes through one-on-one coaching and regular care team meetings. The care team meetings provided a platform for feedback, peer supervision and knowledge exchange between practice nurses. Training and education of practice nurses took place before as well as alongside the intervention. Before the intervention, nurses participated in a 3-day motivational interviewing course and received a one-day RAI-CHA training. Alongside the intervention, nurses received an extra ‘training on the job’ motivational interviewing training, and regularly received education about geriatric themes during care team gatherings.

### Implementation at an organizational level: multidisciplinary team consultations and community networks

Multidisciplinary team consultations (MTCs) were organized with the aim to enhance the coordination between professionals caring for a single patient with complex needs. MTCs were attended by a practice nurse, a primary care physician, the geriatric expert team, a pharmacist and, if present, caregivers involved in the older person’s treatment or care. If relevant and feasible, older people’s partner or family members were invited to join the meeting. Older people were informed of MTC outcomes by the practice nurse. To build community networks, the geriatric expert teams organized meetings with local service providers and organizations. The aim of such meetings was to target the fragmentation of care delivery on a community level by facilitating coordination between professionals and encouraging optimal use of local resources.

### The ACT study

**Figure 1:**
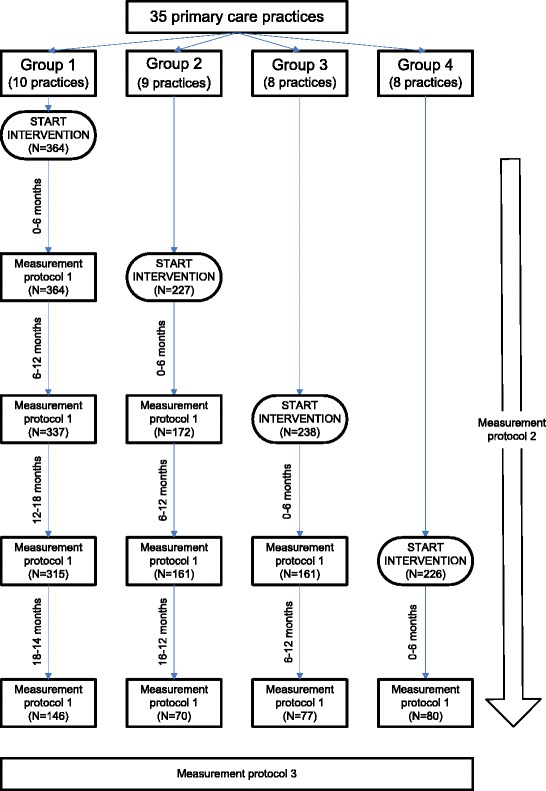
**Flow chart of measurements.** Measurement protocol 1 = coverage (geriatric assessment delivery, care plan delivery, MTC delivery), frequency (geriatric assessment delivery, care plan delivery, MTC delivery); Measurement protocol 2 = frequency (care team meetings, community network meetings), duration (geriatric assessment delivery, care plan delivery, MTC delivery), moderating factor ‘availability of facilitation strategies’. Protocol 3 = moderating factors ‘participant responsiveness’.

The ACT study introduced the Geriatric Care Model to 1147 frail, older patients of 35 primary care practices in two regions of the Netherlands, using a stepped-wedge Randomised Controlled Trial (RCT) design [33]. Frailty was established by a primary care physician using a composite definition of frailty (experiencing one or more limitations in either physical, psychological and/or social areas) [3] and a polypharmacy criterion (5 or more drugs prescribed in the last 3 months); frail older people who scored 3 or higher on the Program on Research for Integrating Services for the Maintenance of Autonomy case-finding tool for disability (PRISMA-7) [35] were eligible for trial entry. As per stepped-wedge RCT protocol, practices in both regions were randomly allocated to four groups (i.e. group 1, consisting of 10 practices; group 2, consisting of 9 practices; group 3 and 4, both consisting of 8 practices), that started working according to the Geriatric Care Model at different times during the trial period (at 0, 6, 12 and 18 months, respectively, see Figure 1). Practice nurses joined the care team (one care team per region) when the primary care practice they worked for started with the intervention. The ACT study received approval by the medical ethics committee of the VU University medical centre.

### Procedures and outcomes

To operationalize implementation fidelity, we first identified ‘active’ components of the Geriatric Care Model (i.e. components of which optimal implementation is theorized as essential in order for the intervention to be successful). On a patient level, we considered the geriatric assessment and the care plans essential; on a care professional level, we considered the care team meetings essential; on organizational level, we considered the MTCs and community network meetings essential. Subsequently, we determined criteria for planned delivery of the essential intervention components. Depending on group allocation, it was planned that older people receive a geriatric assessment at least four (group 1), three (group 2), two (group 3) or one (group 4) times throughout the intervention period, with a maximum of 6 months between the starting time of the intervention and the first assessment and between two subsequent assessments. All geriatric assessments are carried out in the older person’s home using the web-based version of the RAI-instrument, and assessment questions are administered in the order in which they appear. Furthermore, it was planned that each assessment is followed by a care plan, which is discussed with the client in a follow-up home visit and left with the client. Care team meetings are organized regularly by geriatric expert teams, and are attended by all practice nurses. Each primary care practice organizes at least 2 MTCs every 6 months of the intervention, and MTC’s are attended by a practice nurse, a primary care physician, the geriatric expert team, a pharmacist and, if indicated, caregivers involved in the older person’s treatment or care. Community network meetings are organised throughout the intervention period.

Based on the above criteria for delivery, we formulated research questions for each subcategory of adherence based on Carroll’s framework for fidelity, as well as for the moderating factors ‘availability of facilitation strategies’ and ‘participant responsiveness’. In addition, we formulated research questions to investigate other factors that could have moderated adherence (see Table 1). Quantitative data were collected using three measurement protocols (Table 1 and Figure 1). Protocol 1 measured (1) coverage of assessment delivery, care plan delivery and MTC delivery, and (2) frequency of assessment delivery, care plan delivery, and MTC delivery. Protocol 2 measured (1) frequency of care team meetings and community network meetings, and (2) duration of assessment delivery, care plan delivery, and MTC delivery. Protocol 3 measured the moderating factors ‘availability of facilitation strategies’ and ‘participant responsiveness’. Qualitative data were collected alongside the intervention to assess the intervention’s content and other factors that could have moderated adherence.

**Table 1:** **Research questions per subcategory of adherence and assessed moderating factor**

**Adherence subcategory**	**Intervention component**	**Research question**	**Measurements**	**Data source or procedure**
Coverage	Geriatric assessment	What proportion of participants received at least one assessment?	6, 12, 18 and 24 months after start of intervention	RAI-CHA database
	Care plan	What proportion of participants received a care plan after each assessment?	6, 12, 18 and 24 months after start of intervention	Digital copies care plans
	MTCs	What proportion of primary care practices delivered at least two MTCs?	6, 12, 18 and 24 months after start of intervention	Geriatric expert team logbooks
				MTC reports
Frequency	Geriatric assessment	How often were assessments delivered?	6, 12, 18 and 24 months after start of intervention	RAI-CHA database
	Care plan	How often were care plans delivered?	6, 12, 18 and 24 months after start of intervention	Digital copies care plans
	Care team meetings	How many care team meetings took place?	Alongside the intervention	Geriatric expert team logbooks and minutes of care team meetings
	MTCs	How often were MTCs delivered?	6, 12, 18 and 24 months after start of intervention	Geriatric expert team logbooks
				MTC reports
	Community network meetings	How many community network meetings took place?	Alongside the intervention	Geriatric expert team logbooks
Duration	Assessment Care plan MTCs	What was the average duration (in minutes) of activities involving the assessment/care plan/MTC?	Alongside the intervention	Time sheets practice nurses
Content	Geriatric assessment Care plan Care team meetings	How were the geriatric assessments, care plans and care team meetings delivered?	Alongside the intervention	Semi-structured interviews practice nurses and geriatric expert team members
	MTCs	How were the MTCs delivered, and who attended the MTCs?	Alongside the intervention and at the end of the intervention period	Semi-structured interviews practice nurses, geriatric expert team members and primary care physicians
				Geriatric expert team logbooks
	Community network meetings	How were the community network meetings delivered?	Alongside the intervention	Semi-structured interviews with geriatric expert team members
**Moderating factor**				
Availability of facilitation strategies	Educational sessions	How many educational session took place?	At the end of the intervention period	Geriatric expert team logbooks
				Minutes of team meetings
	Pre-intervention training sessions	How many practice nurses attended the pre-intervention training sessions?	At the end of the intervention period	Geriatric expert team logbooks
				Minutes of team meetings
	Nurses’ experiences with educational and training sessions	What were practice nurses’ experiences with the educational and training sessions?	Alongside the intervention	Semi-structured interviews practice nurses
Participant responsiveness	Satisfaction Involvement	How satisfied were practice nurses and primary care physicians with the intervention, and how involved did they feel?	At the end of the intervention period	Nurses/physicians were asked to rate their satisfaction and involvement on a 1-10 scale.
Other moderating factors		What other factors modified adherence?	Alongside the intervention	Semi-structured interviews practice nurses, geriatric expert team members and primary care physicians

MTC = Multidisciplinary Team Consultation; RAI-CHA = Resident Assessment Instrument – Community Health Assessment.

Since the starting time of the intervention varied per study participant and primary care practice, we defined ‘start of the intervention’ on a participant and care professional level as the date an older person was invited for a first assessment, and ‘start of the intervention’ on a organisational level as the date the first patient of a practice was invited for a first assessment. The total intervention period was determined as follows: starting time plus 24 months (group 1); starting time plus 18 months (group 2), starting time plus 12 months (group 3); starting time plus 6 months (group 4).

### Adherence measurements

Coverage of geriatric assessment delivery was measured per 6-month interval, and calculated by dividing the number of older people who received at least one assessment by the total number of older people intended to receive at least one assessment. Coverage of care plan delivery was measured per 6-month interval, and calculated by dividing the total number of older people who received a care plan after each assessment by the total number of assessments. Coverage of MTC delivery was measured per 6-month interval, and calculated by dividing the number of primary care practices that achieved the intended amount of MTCs by the total number of primary care practices that participated in the intervention. One MTC corresponded with one patient discussed. Since the number of care team meetings and community network meetings was not specified before the start of the intervention, we did not include these in the assessment of coverage.

Frequency of geriatric assessment delivery was measured per 6-month interval, and calculated by dividing the total number of assessment deliveries by the number of intended assessment deliveries. Frequency of care plan delivery was measured per 6-month interval, and calculated as the total number of care plan deliveries divided by the number of intended care plan deliveries. Frequency of MTC delivery was measured per 6-month interval, and calculated as the total number of MTCs that were organized by primary care practices divided by the intended number of MTC’s per primary care practice. Since no intended 6-month delivery was formulated at the start of the intervention, frequency of care team meetings and community network meetings was calculated as the total number of care team and network meetings that took place during the intervention period.

To determine duration of the intervention components, practice nurses (N = 14) used time tracking. Duration of assessment delivery was calculated as the average amount of time (in minutes) it took nurses to carry out one assessment, including pre-assessment and post-assessment activities. Duration of care plan delivery was calculated as the average amount of time (in minutes) it took for a practice nurse to write a care plan, discuss it with a primary care physician, and discuss it during a second follow-up home visit. Duration of MTCs was calculated as the average amount of time (in minutes) it took to discuss one person in an MTC. The duration of care team meetings and network meetings was not included in the assessment.

### Moderating factors measurements

To assess the moderating factor *availability of facilitation strategies*, we determined how many educational sessions were organized by the geriatric expert team. In addition, we determined how many practice nurses attended the pre-intervention training sessions. To assess *participant responsiveness*, practice nurses and primary care physicians were asked to rate their satisfaction and involvement with the intervention on a scale of 1 to 10 (1 representing minimal satisfaction/involvement, 10 representing maximum satisfaction/involvement).

### Qualitative data collection

To investigate content and find factors that moderated adherence, we used qualitative data collection. Semi-structured interviews with practice nurses (N = 15), geriatric expert team members (N = 3) and primary care physicians (N = 10) were conducted alongside the intervention. We included the following topics (per intervention component): order in which protocol activities are carried out; work practices, deviations from protocol, component duration, perceived quality and usefulness of the protocol, barriers and facilitators to carrying out the protocol as intended, self-perceived competence to carry out the protocol as intended, points of improvement. We took a cyclic approach: when a new topic emerged from an interview, it was added to the topic list of the next interview. All interviews were audio recorded and transcribed. To enhance the quality of the qualitative data, respondents were asked to approve a summary of the interview (member check). In addition, the researcher who carried out the interviews (MM) kept a research log in which she reflected on methodological decisions and her own role in the research process.

### Analysis

Quantitative data were analyzed with descriptive statistics using SPSS Statistics 20. Study drop out was accounted for on a patient and primary care practice level. Since adherence was calculated per 6-month interval, older people who had dropped out were excluded from analysis for the whole 6-month interval in which the drop-out occurred. For instance, a drop out at 16 months implied exclusion from analysis from 12 months onwards. One primary care practice in group 4 was excluded from analysis due to drop out prior to its designated starting time. We assessed coverage of assessment and care plan delivery on a practice nurse level to allow for an investigation of inter-nurse variance. We used Spearman’s rank correlation coefficient to investigate the correlation between participant responsiveness rates of practice nurses and family physicians and their average coverage rates of the key intervention components (geriatric assessment, care plan and MTC), using the criteria for the interpretation of the effect size proposed by Ferguson (weak, moderate, strong) [36].

Qualitative data were analyzed using a framework approach, a method of analysis in which some of the research questions are predetermined. Two researchers (MM and KL) analyzed the interviews separately and discussed their results to achieve intersubjective agreement. First, both researchers familiarized with the data. Second, they applied labels to the data and arranged the data according to broader themes.

## Results

### Adherence to assessments and care plans

Coverage of geriatric assessment delivery in group 1, 2 and 3 was high in the first 6 months of the intervention (ranging from 83.2% to 91.3%), but declined in the following months. In group 4, first 6-month coverage was the lowest (79.8%). Table 2 shows coverage of geriatric assessment delivery per tranche and per 6-month interval. Average coverage rates between practice nurses varied: 13 nurses reached an average of 70-100%, and 4 nurses reached an average of 40-70%. Frequency of geriatric assessment delivery was overall the highest in group 1, with a peak in the first 6 months (103.0%). In all four groups, frequency never fell below 72% (see Table 3). It took practice nurses an average of 76 minutes to carry out the assessment. The average time spent on pre-assessment and post-assessment activities (e.g. administration) was 43 minutes. All nurses regularly or always used a paper version of the assessment instrument instead of the online version, and four nurses said that they did not always ask the RAI-CHA questions as intended. Two nurses indicated that when an older person visited their office for something not related to the intervention, assessments were sometimes carried out there instead of in the clients’ home.

**Table 2:** **Coverage of key intervention components**

	**Intervention period**			
	**0-6 months**	**6-12 months**	**12-18 months**	**18-24 months**
	**N delivered/N planned (%)**	**N delivered/N planned (%)**	**N delivered/N planned (%)**	**N delivered/N planned (%)**
*Group 1*				
Geriatric assessment	329/364 (90.4)	227/337 (67.4)	181/315 (57.5)	212/304 (69.7)
Care plan	318/331 (96.1)	231/234 (98.7)	186/198 (93.9)	168/216 (77.8)
MTC	1/10 (10)	3/10 (30)	0/10 (0)	5/10 (50)
*Group 2*				
Geriatric assessment	157/172 (91.3)	146/161 (90.7)	132/158 (80.0)	
Care plan	155/157 (98.7)	130/146 (89.0)	121/136 (89.0)	
MTC	0/9 (0)	2/9 (22.2)	2/9 (22.2)	
*Group 3*				
Geriatric assessment	134/161 (83.2)	114/155 (73.5)		
Care plan	126/134 (94.0)	111/118 (94.1)		
MTC	1/8 (12.5)	4/8 (50)		
*Group 4*				
Geriatric assessment	103/129 (79.8)			
Care plan	77/103 (74.8)			
MTC	0/7 (0)			

MTC = Multidisciplinary Team Consultation.

**Table 3:** **Frequency of key intervention components**

	**Intervention period**			
	**0-6 months**	**6-12 months**	**12-18 months**	**18-24 months**
	**N delivered**	**N delivered**	**N delivered**	**N delivered**
*Group 1*				
Geriatric assessment	375/364 (103.0)	263/337 (78.0)	227/315 (72.1)	232/304 (76.3)
Care plan	351/375 (93.6)	257/263 (97.7)	231/227 (101.7)	181/232 (78.0)
MTC	3/20 (15.0)	10/20 (50.0)	2/20 (10.0)	22/20 (110.0)
*Group 2*				
Geriatric assessment	171/172 (99.4)	163/161 (101.2)	144/158 (91.1)	
Care plan	169/171 (98.8)	144/163 (88.3)	129/144 (89.6)	
MTC	0/18 (0.0)	6/18 (33.3)	6/18 (33.3)	
*Group 3*				
Geriatric assessment	148/161 (91.9)	120/155 (77.4)		
Care plan	139/148 (91.9)	113/120 (94.2)		
MTC	2/16 (12.5)	9/16 (56.3)		
*Group 4*				
Geriatric assessment	106/129 (82.2)			
Care plan	79/106 (74.5)			
MTC	1/16 (6.3)			

MTC = Multidisciplinary Team Consultation.

In group 1, coverage of care plan delivery stayed between 99% and 94% in the first 18 months of the interventions but dropped to 78% in the last 6 months. In group 2 and 3, coverage stayed between 89% and 99% throughout the intervention period; group 4 showed the lowest coverage of care plan delivery (74.8%) (Table 2). Besides one nurse in group 4 whose coverage remained 0%, we found little variation between nurses: only 3 nurses reached an average of 60-80%, while the remaining nurses achieved averages up to 100%. In group 1-3, frequency of care plan delivery did not fall below 90% until the last 6 months of the intervention; in group 3, it never fell below 90%. Frequency of care plan delivery was lowest in group 4 (74.5%) (see Table 3). Nurses spent an average of 37 minutes writing a participant’s care plan and a little over half an hour discussing the care plan during the follow-up visit. Care plans and follow-up visits were not always carried out as intended. Some care plans did not include the intended information (i.e. they were incomplete), and some care plans did not get written at all. In addition, follow-up visits did not always take place. Seven nurses did not always leave the care plans with their clients.

### Adherence to MTCs, care team meetings and community network meetings

In groups 1 to 3, MTC coverage started low but improved over time, with coverage in group 1 and group 3 reaching 50% in the last 6 months of the intervention (Table 2); in group 4, none of the primary care practices organized the MTCs as intended. Average coverage varied between individual practices: 13 achieved a coverage between 50-100%, 5 achieved a coverage of 0-50%, and 17 practices never carried out an MTC as intended. Group 1 contained almost 40% of the practices with a 50-100% coverage rate, whereas group 4 contained 40% of practices with a 0% coverage rate. Frequency of MTC delivery varied between and across the four groups. Generally, frequency increased over time. Practices in group 1 organized three times as much MTCs as practices in group 2 and 3. In group 4, only one MTC was organized during the designated intervention period. Preparing and carrying out MTCs took practice nurses almost 40 minutes per participant. MTCs were always attended by the primary care physician, the attending practice nurse, both members of the geriatric expert team and a pharmacist. Almost half of the MTCs were joined by other care professionals (such as a home care nurse or a physiotherapist) or the client’s partner or family members. Most MTCs involved discussing clients with complex care needs (17 meetings), severe pain (7 meetings), cognitive or mood problems (5 meetings), poor support networks (3 meetings) and rejection of care behaviour (2 meetings). In addition, some meetings were specifically aimed at coordinating care delivery (6 meetings) or revise medication (2 meetings).

Over the course of the intervention, the geriatric expert teams in the two regions organized a total of 45 team meetings. During the meetings, nurses discussed complex cases, consulted the geriatric expert team and received project updates. In addition, the geriatric expert teams organized 10 community network meetings. Network meetings were attended by professionals participating in the ACT study, local government representatives and care organizations active in the community.

### Moderating factors: availability of facilitation strategies and participant responsiveness

Throughout the intervention period, geriatric expert team members organised 18 educational sessions, 9 of which featured short seminars by care professionals of regional care organizations aimed at educating practice nurses about available community resources. Session and seminar topics ranged from urinary incontinence and pain to nutrition and dementia. Of the 21 practice nurses, 20 nurses completed the 3-day training program ‘motivational interviewing’, and 17 nurses attended the RAI-CHA assessment training. All practice nurses said that they highly valued the motivational interview training, but did not always evaluate the RAI-CHA training as sufficiently educational or useful. Most nurses did not feel sufficiently confident about their assessment skills when they started with the intervention, and indicated that they would have preferred a more intensive assessment training schedule.

On average, practice nurses rated their involvement in the ACT study with a 7.1 (range 2-10) and their satisfaction with their work with a 6.5 (range 3-8). Associations between nurses’ satisfaction and involvement and their achieved coverage rates were weak and not significant. Primary care physicians rated their involvement with a 6.7 (range 2-9) and their satisfaction with a 6.3 (range 3-9). Association between physician involvement and satisfaction and their achieved MTC coverage rates were weak and moderate, respectively.

### Other moderating factors

Results of qualitative investigation showed several factors that may have moderated adherence of the various intervention components.

Adherence to geriatric assessment delivery was influenced by factors at a participant, practice nurse and resource level. First, older people included in the ACT-study were not always available for an assessment due to long-term hospital stays, out-of-town holidays and episodes of illness or weakness. Second, nurses’ preference to carry out the assessment on paper instead of online was the result of limited network services, and since nurses generally preferred a natural conversation with a client over a structured interview, they often did not ask assessment questions in their intended order. Finally, the time it took to carry out an assessment was impacted by nurses’ own competencies (four nurses mentioned that their competencies increased over the course of the intervention, which they said reduced overall assessment time) and by the extent to which older people wished to share stories and experiences.

Adherence to care plan delivery was mostly influenced by factors at a patient and a practice nurse level. Some nurses indicated that whether they wrote a care plan and scheduled a follow-up visit depended on the amount of time pressure they experienced and on the assessment outcomes (i.e. they abstained from writing a care plan and following up when an assessment had yielded no health or care needs). One nurse in group 4 never adopted the care plan method, because she felt that the method was not tailored to her needs (for instance, it could not be integrated into a digital patient information system) and had developed her own way of communicating assessment outcomes with clients and colleagues. Nurses also mentioned several reasons why they did not always leave the care plan with their client as intended: one nurse believed the plan was redundant in the presence of existing medical documentation, others said that older people refused to accept the plan because it contained sensitive information, because it was of little or no value to them, or because they had no health or care needs.

Adherence to MTC delivery was mostly influenced by factors at a participant and primary care physician level. Results show that an MTC was cancelled twice due to the older person passing away unexpectedly or to a resolving of the issues that initially urged the MTC. Four primary care physicians indicated that they believed MTCs were redundant, too time consuming or inefficient. Three practice nurses mentioned that physicians diverted their efforts to organize an MTC, and geriatric team members experienced that it took a while before physicians got used to the idea of attending an MTC.

Finally, factors on a practice nurse and geriatric team level played a role in the adherence to care team meetings and community network meetings. Care team meetings were valued by nurses, who mentioned that they appreciated the opportunity for peer-to-peer practical information exchange about intervention procedures and tasks, such as working with the RAI-assessment instrument and care plans. We found that organizing community network meetings was challenging for geriatric teams for several reasons. One team member mentioned the challenge of identifying and locating network partners, another team member was unsure about her own role in the networking process. Moreover, it was pointed out that networking was hindered by the decentralization and fragmentation of target organisations and providers.

## Discussion

Using Carroll’s framework, we assessed implementation fidelity of the geriatric care model, a chronic care model for frail, older people who live at home.

Overall, we found that the adherence to geriatric assessment and care plan delivery was high: especially in the first 6 months after the start of the intervention, a large share of participants received the geriatric assessments and care plans as planned and as often as planned. However, over time, adherence of these two components decreased. This gradual decrease may be partly explained by ‘delayed delivery’ of the intervention, i.e. the number of months between two subsequent geriatric assessments exceeded the intended six. While practice nurses eventually delivered such ‘delayed assessments’, assessments increasingly fell beyond the intended time frame. As a consequence, we classified them as ‘not delivered as intended’. Delayed delivery likely occurred as a consequence of early start-up issues that impeded initial implementation.

In group 4, first 6-month coverage of geriatric assessment delivery and care plan delivery was lower than in the other three groups. In the case of assessment delivery, the cause of this difference remains unclear – we found no obvious inter-group variations in adherence or moderating factors on a professional, patient or practice level. In the case of care plan delivery, the difference could be explained by the fact that one nurse in group 4 never adopted the care plan method, a deviation that may be associated with a lack of involvement in the intervention: the same nurse never attended a care team meeting, and infrequently responded to emails or phone calls.

As opposed to the geriatric assessments and care plans, overall first 6-month adherence to MTC delivery was low but increased over time. Our data suggest that the longer a practice was exposed to the intervention, the higher the overall coverage. This finding may be explained by the fact that in the Netherlands, structural multidisciplinary encounters are a relatively new phenomenon in primary care, and at the time of the intervention few examples existed as to how to integrate MTCs into daily practice. In addition, organising and carrying out an MTC is complex, a component characteristic known to be associated with lower fidelity [20].

Care team meetings were organized as intended and highly valued by the participants. Since most of the time nurses performed their intervention activities alone, they appreciated the interactive nature of the meetings, and the opportunity to give and receive direct peer feedback. Community network meetings were more challenging to implement. Geriatric expert team members experienced barriers in their efforts to build and sustain professional networks. These challenges may be partly explained by Rogers’ ‘Diffusion of Innovation’ theory, that suggests that individuals who are more interpersonally connected within an organisational system are more likely to adopt an innovation [37]. Expert team members were motivated to set up networks because they were in the core group of Geriatric Care Model implementers, whereas potential network partners were on the group periphery and thus less likely to change their routines. In addition, implementation of network meetings may have been challenging because geriatric expert team members had limited previous networking experience. Finally, the decentralisation issues that urged the meetings may also have hindered the meetings’ actualisation in practice.

Our results show that factors at a participant, care professional, organizational and resource level may have moderated adherence to the various intervention components. We found that there were large variations in the extent to which nurses and physicians felt satisfied and involved with the intervention. These variations were most likely determined by individual factors: previous research suggests that nurse job satisfaction is often influenced by work attitude, job stress and nurse-physician interaction, among others [38,39], and that physician job satisfaction can be influenced by factors such as personality, workload and recognition [40,41]. Involvement and satisfaction of both nurses and physicians was only weakly or moderately associated with coverage of geriatric assessment delivery and care plan delivery, which suggests that there was little influence of participant responsiveness on nurses’ or physicians’ individual achievements. Furthermore, the pre-intervention RAI-CHA training program did not always succeed in giving practice nurses the knowledge and skills to implement the assessment as intended, which may have affected the adherence subcategories duration and content. Future implementors of the Geriatric Care Model should secure that training programs are tailored to the learning needs of the users.

### Measuring CCM implementation fidelity

Research papers that report outcomes of the implementation of CCM-based interventions in randomised controlled trials do not always include a thorough investigation of the degree of implementation. However, researchers who do undertake such an investigation typically opt to develop their own conceptual and methodological framework due to a lack of standardized methods. For instance, when Pearson et al. assessed CCM implementation of 42 organisations, they distinguished between fidelity and intensity, defining fidelity in terms of alignment of observed change activities with the various CCM elements and intensity in terms of quantity and depth [42]. Haggstrom et al. used surveys based on qualitative interviews to assess the implementation of the CCM among community health centers [43], while Hroscikoski et al. used a qualitative, comparative case study design to evaluate CCM implementation in a large health care organisation [44]. The differences in operationalization and methodology hinders between-program comparisons of implementation parameters, limiting our understanding of what aspects of a program’s content and context contribute to its potential to achieve a high degree of implementation. We therefore recommend researchers who aim to assess the implementation of a CCM-based program to chose an existing framework, such as Carroll’s, and adjust it according to the program’s particularities.

### Study limitations

Several study aspects may have limited our insight in the implementation fidelity of the Geriatric Care Model. First, there were limitations to our investigation of adherence. Time constraints prevented practice nurses from consistently tracking the duration of their activities, which restricted the collection of longitudinal data on intervention duration. The intended delivery of care team meetings and community network meetings was unspecified, which caused us to measure adherence to these components only at the end of the intervention period. Second, due to the complexity of the intervention, we investigated only two of the six factors that moderate fidelity proposed by Hasson et al. [32]. Investigation of each of the moderating factors was subject to limitations. Participant responsiveness was measured only at the end of the intervention and using a single scale. Collecting more data on responsiveness, for instance through qualitative interviews, could have deepened our understanding of the way responsiveness influenced adherence. A longitudinal investigation of participant responsiveness could have given us insight in the way in which responsiveness developed throughout the intervention; in addition, it could have enhanced our understanding of the influence of responsiveness on adherence over time. Our understanding of the moderating influence of facilitation strategies would have benefitted from an investigation of attendance at the education sessions; however, data collection was impaired due to incomplete records and attendance sheets.

### Nonadherence in real world settings

It is recognized that interventions cannot always be fully implemented as intended in the real world [20,45]. This is even more true for multicomponent interventions, whose complex nature increases the scope for variations in delivery [20]. Some authors argue therefore that, in order to accommodate successful implementation in non-experimental settings, it is necessary to tailor program content to local conditions. Others share this opinion, but state that low fidelity of ‘active’ intervention components (i.e. key program elements considered essential to intervention success) can result in interventions not achieving the intended effects [20]. Although the professionals who carried out the Geriatric Care Model performed their activities within the framework of a protocol, they were simultaneously encouraged to adjust their activities to the local primary care context. By making these adjustments, professionals may have deviated from the initial intervention protocol. Since this protocol served as a template for the fidelity assessment, deliberate deviations may have consequentially been interpreted as nonadherence (i.e. such activities may have been characterized as ‘not implemented as intended’). Hypothetically, we are therefore confronted with a paradox: while it is likely that poor adherence or nonadherence contributed to a lower overall estimation of implementation fidelity, it could at the same time have improved actual implementation of the geriatric care model in the real world. This paradox eventually complicates the use of outcomes of fidelity assessment to explain findings of effectiveness evaluation.

## Conclusion

This study is the first to investigate implementation fidelity of the Geriatric Care Model, a comprehensive care model for frail, older people in a primary care setting based on the Chronic Care Model. We found that, despite variations in nurses’ level of participant responsiveness, the adherence to geriatric assessment delivery and care plan delivery was high, and that adherence to multidisciplinary team meetings was initially low but increased over time. In addition, we found that the level of adherence varied between professionals, which most likely can be attributed to professional’s individual characteristics and circumstances.

We demonstrated that a longitudinal investigation of adherence can contribute to our understanding of the ways in which adherence develops over time, and therefore recommend that researchers who aim to investigate fidelity of complex interventions measure all essential intervention components and moderating factors at multiple times during the intervention period. Researchers who carry out such an investigation should keep in mind that factors on a professional level, such as time restraints and lack of involvement, could complicate data collection and subsequently influence results. In addition, researchers should be aware of the fact that the real life setting increases the likelihood that professionals use nonadherence as a means to tailor the intervention to local conditions, which may challenge their ability to relate outcomes of fidelity assessments to conclusions about program feasibility or effectiveness.
